# Integrated Full-Length Transcriptome and RNA-Seq to Identify Immune System Genes from the Skin of Sperm Whale (*Physeter macrocephalus*)

**DOI:** 10.3390/genes12020233

**Published:** 2021-02-05

**Authors:** Daling Wang, Ying Li, Reyilamu Aierken, Qi Kang, Xianyan Wang, Qianhui Zeng, Zhichang Fan, Yu Zhen, Liyuan Zhao

**Affiliations:** 1 College of Environmental Science and Engineering, Ocean University of China, Qingdao 266100, China; wangdaling@stu.ouc.edu.cn (D.W.); liying123hh@163.com (Y.L.); 2 Laboratory of Marine Biology and Ecology, Fujian Provincial Key Laboratory of Marine Ecological Conservation and Restoration, Third Institute of Oceanography, Ministry of Natural Resources, Xiamen 361005, China; rylm@foxmail.com (R.A.); wangxianyan@tio.org.cn (X.W.); zengqianhui@tio.org.cn (Q.Z.); 3 Key Laboratory of Marine Environment and Ecology, Ocean University of China, Ministry of Education, Qingdao 266100, China; 4 State Key Laboratory of Cellular Stress Biology, School of Life Sciences, Xiamen University, Xiamen 361102, China; kangqix@126.com; 5 Ocean College, Shandong University, Wehai 264209, China; 6 School of Pharmaceutical Sciences, Xiamen University, Xiamen 361102, China; fzhichang@126.com

**Keywords:** NOD-like receptor (NLR)-signaling pathway, NOD1, sperm whale, transcriptome

## Abstract

Cetaceans are a group of secondary aquatic mammals whose ancestors returned to the ocean from land, and during evolution, their immune systems adapted to the aquatic environment. Their skin, as the primary barrier to environmental pathogens, supposedly evolved to adapt to a new living environment. However, the immune system in the skin of cetaceans and the associated molecular mechanisms are still largely unknown. To better understand the immune system, we extracted RNA from the sperm whale’s (*Physeter macrocephalus*) skin and performed PacBio full-length sequencing and RNA-seq sequencing. We obtained a total of 96,350 full-length transcripts with an average length of 1705 bp and detected 5150 genes that were associated with 21 immune-related pathways by gene annotation enrichment analysis. Moreover, we found 89 encoding genes corresponding to 33 proteins were annotated in the NOD-like receptor (NLR)-signaling pathway, including *NOD1*, *NOD2*, *RIP2*, and *NF-κB* genes, which were discussed in detail and predicted to play essential roles in the immune system of the sperm whale. Furthermore, *NOD1* was highly conservative during evolution by the sequence comparison and phylogenetic tree. These results provide new information about the immune system in the skin of cetaceans, as well as the evolution of immune-related genes.

## 1. Introduction

Cetaceans (whales, dolphins, and porpoise) are a group of secondary aquatic mammals whose ancestors returned to the ocean from land and then gradually evolved into the dominant group of marine mammals approximately 53 million years ago [1], which is one of the most dramatic events in the history of biological evolution. When cetaceans entered the ocean from land and rapidly radiated in different waters around the world, their immune systems were likely attacked by many kinds of pathogenic microorganisms in different environments. Therefore, cetaceans may be ideal models for studying the evolution process of vertebrate immune genes and related driving mechanisms. Sperm whale (*P. macrocephalus*) belongs to the first branching lineage of extant Odontoceti, family Physeteridae [2]. They are the largest living toothed whale and are found in all oceans of the world [3,4]. Sperm whales are also the deepest diving whales, who can dive to a depth of 3000 m and stay underwater for at least 138 min [5]. These characteristics imply they may have unique adaptations and an important phylogenetic position [6,7]. Till now, the sperm whales have become endangered species and have been listed in the First-Order of the National Key Protected Wild Aquatic Animals List in China and the Convention International Trade in Endangered Species of Wild Fauna and Flora (CITES), a multilateral treaty to protect endangered plants and animals. Moreover, since 2012, it has been included in the Red List of Threatened Species by the International Union for the Conservation of Nature and Natural Resources (IUCN) [8].

There are more pathogenic organisms (e.g., bacteria, fungi, and parasites) in aquatic environments than on land [9,10]. Cetaceans have undergone morphological and physiological changes to adapt to the complete aquatic lifestyle [11,12,13]. Therefore, much research has focused on cetacean immunity. For example, natural killer cell activity in peripheral blood mononuclear cells (PBMC) was shown to play a significant role in defending against viral infections in beluga whales (*Delphinapterus leucas*) [14]. The lymphoid organs of cetaceans were found to have unique anatomical structures compared with those of other mammals, including complex lymphoepithelial larynx glands that may be an adaptation to the marine environment [15]. Immune-related MCH II (+) cells were found to be dendritic in the skin of bottlenose dolphins (*Tursiops truncatus*) [16]. Additionally, differentially expressed immune-related genes have been detected ex vivo in the skin of bottlenose dolphins treated with different concentrations of pollutants [17]. Many genes involved in the immune response and adaptive evolution of cetaceans have been discovered by evolutionary analysis [18]. Moreover, some genes in the Toll-like receptor (TLR)-signaling pathway have been studied in the evolution of cetaceans [19,20].

NOD-like receptors (NLRs) are a type of intracellular pattern-recognition receptors (PRRs), which can mediate innate response and are associated with resistance to pathogenic microorganisms, and have roles in protecting against infection and cell damage [21,22]. As an important member of innate immunity effector molecules, the intracellular NLR family contains more than 20 members in mammals, which plays a vital role in the identification of intracellular ligands [23]. Certain NLRs have been confirmed to be involved in pathogen clearance and inflammasomes [24,25]. In mammals, the nucleotide-binding oligomerization domain (NOD) molecules, NOD1 and NOD2, which are cytoplasmic surveillance proteins, can sense the cytosolic presence of the bacterial peptidoglycan fragments that escaped from endosomal compartments, driving the activation of NF-κB and MAPK, cytokine production and apoptosis [26,27]. However, the NLR-signaling pathway has never been reported in cetaceans.

Skin is one of the major obstacles to the external environment, which plays a vital role in resisting pathogens. Compared with short-read sequencing, the methodological advantages of PacBio isoform sequencing (Iso-Seq) include better integrity in sequencing both the 5′ and 3′ ends of the full-length cDNA molecules and higher accuracy in generating isoform-level transcripts. However, the expression level of the transcripts (FPKM) can be better calculated by Illumina sequencing. In this study, we performed a combination study of PacBio Iso-Seq and de novo short-read RNA-seq to generate high-quality, full-length transcript data of the skin of the sperm whale. Subsequently, the seven public databases were used to systematically annotate these full-length transcripts. Many transcripts of the sperm whale were annotated in immune-related pathways in the KEGG database. Moreover, the NOD-like receptor-signaling pathway has the largest number of genes annotated in all innate immune pathways. Finally, the structure and the phylogenetic tree analysis has been performed on the sperm whale NOD1 to help us understand the evolutionary process of *NOD1* genes in cetacean and different vertebrate species. Our research will provide silicon predictions for future research on the immune system of cetaceans.

## 2. Materials and Methods

### 2.1. Animal and Tissue Collection

The sperm whale (*P. macrocephalus*) used in this study was discovered alive and trapped in fishing nets in the waters of Daya Bay (Guangdong, China) on 12 March 2017. It died on 15 March after an approximately 72 h rescue attempt. It has been recognized that the most likely cause of death for this sperm whale was prolonged entanglement leading to malnutrition/starvation, exhaustion, dehydration, and death [28]. The dead animal was salvaged and necropsied according to standard protocols. It was about 10.78 m in length and 14.18 t in weight, and, after an anatomical investigation, it was found to be a female sperm whale with a fetus. This sperm whale was necropsied just after death. The skin of the female was collected with a stainless steel scalpel during the necropsy and preserved in 1 mL RNA-later solution (Applied Biosystems, Warrington, UK) and stored at 4 °C for 24 h and then transferred to −80 °C until RNA extraction.

### 2.2. RNA Extraction and Quantification

**Figure 1:**
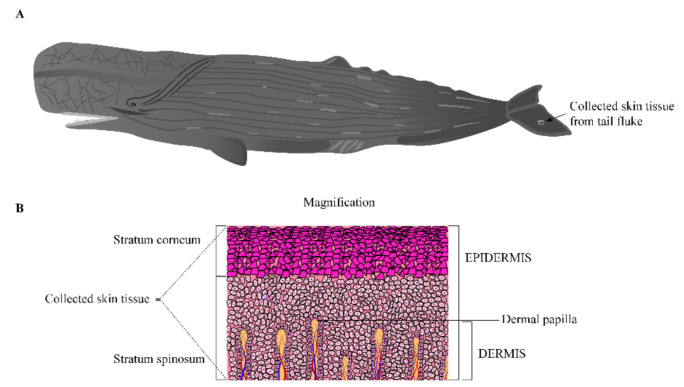
Schematic diagram of the collected skin tissue sample from the sperm whale. (**A**) The location of the collected skin tissue sample from the sperm whale. (**B**) The layers structure of the collected skin tissue sample.

Total RNA was isolated from a piece of skin tissue from the sperm whale’s tail using TRIzolTM reagent (Thermo Fisher Scientific, Waltham, MA, USA) according to the manufacturer’s instruction. The schematic diagram of the collected skin tissue sample from the sperm whale in Figure 1A,B. The skin tissue sample contained epidermis and dermis without hypodermis and blubber. We used an RNA 6000 nano kit and an Agilent 2100 bioanalyzer (Agilent Technologies, Santa Clara, CA, USA) to evaluate RNA quality. The RNA quality criteria for the RNA samples were RIN ≥ 7.0, or RIN was close to 7.0, and 1.8 < OD260/280 < 2.2. The qualified total RNA was further used for Illumina library construction, PacBio iso-seq library construction, and subsequent analysis, respectively.

### 2.3. PacBio Iso-Seq Library Preparation and Sequencing

Optional poly-A selection was performed on the qualified total RNA, and the poly (A) RNA was separated with PuristTM Kit (Ambion, Inc, Austin, TX, USA) according to the manufacturer’s instructions. Subsequently, the poly (A) RNA was reverse transcribed using a Clontech SMARTer PCR cDNA synthesis kit (Clontech, Mountain View, CA, USA) to synthesize the first-strand cDNA. After PCR optimization, a large-scale PCR was performed to synthesize second-strand cDNA. To ensure that the long low-abundance transcriptome fragments can be sequenced adequately, we used the BluePippintm system (Sage Science, Beverly, MA, USA) to select the size of the cDNA fragments. Then, the > 4 kb cDNA fragments were mixed with the no size selection library to form the combined SMRTbell library that was used for single-molecule real-time (SMRT) sequencing on a PacBio Sequel system.

### 2.4. RNA-seq Library Preparation and Sequencing

Magnetic beads with oligo (dT) were used to enrich the poly(A) mRNA to process the total RNA; an appropriate amount of interrupting reagent was added to the obtained mRNA at high temperature. We used the interrupted mRNA fragment as a template to synthesize the first-strand cDNA, then configured the second-strand synthesis reaction system to synthesize the second-strand cDNA. After this, we used Ampure XP beads to purify and recover the second-strand cDNA, repaired the sticky ends, and added the “A” base to the 3′ end of the cDNA. The adapter was connected to the cDNA, fragment size was selected, and PCR amplification was performed following the manufacturer’s instructions. Subsequently, quality inspection on the constructed library was performed using the Agilent 2100 Bioanalyzer and ABI StepOnePlus real-time PCR system. Sequencing was performed on the Illumina HiSeq X Ten.

### 2.5. Bioinformatics Analysis of PacBio Data and RNA-seq Data

The raw full-length Iso-seq data were processed following the Iso-seq standard protocol (SMRT Analysis 2.3) on the PacBio SMRT sequencing platform. In our project, The RNA that was extracted from the skin sample was sequenced on the PacBio Sequel platform, and PacBio Iso-seq libraries (0–5 kb) were constructed. During the sequencing reaction, the data that retained the inserts of the sense strand and antisense strand, the 3′ and 5′, joints and the SMRT linker sequence were called polymerase reads, which were subsequently delinked, sequenced repeatedly, clustered and corrected, to get different lengths of reads of insert (ROIs). After processing ROIs under the parameter settings, the minimum sequence was set to 300 bp, and the phmmer algorithm in primer detection was set to 10. ROIs were classified into full-length non-chimeric (FLnc), non-full-length (nFL), chimeric, and short reads. The ROIs were considered to be full-length reads if 5′ and 3′ end linkers and polyA were detected simultaneously. The interactive clustering and error correction (ICE) algorithm was used to predict the isoforms of the full- length non-chimeric, assigning it to clusters, and then the Quiver program was used to correct them into consensus if the cluster contains enough full-length and non-full-length coverage. Fragments were considered as high-quality isoforms when the Quiver quality value was >0.95 in the libraries; others were considered as low-quality isoforms. After the sequence clustering and correction, the high-quality sequences in each library were merged, and redundant sequences were removed to obtain the final transcriptome.

We used SOAPnuke, a filtering software developed by Beijing Genomics Institute (BGI), to count RNA-Seq reads. Trimmomatic was used to remove sequences contained in the adaptor, reads with unknown base N content > 5%, and low-quality reads. We used the Trinity system to perform de novo assembly on clean reads (removing PCR duplicates to improve assembly efficiency) and used Tgicl to cluster the assembled transcripts and redundancy to obtain UniGenes [29]. Bowtie2 program was used to align the UniGenes with the full-length transcriptome of sperm whale skin as reference gene sequences, and finally, the RSEM software package was used to calculate the expression levels of genes and transcripts [30,31]. Those raw data generated were deposited into the National Center for Biotechnology Information (NCBI) database under the accession number SRR13038369 (full-length transcriptome) and SRR13024481 (RNA-Seq transcriptome).

### 2.6. Quantification and Annotation of Gene Expression Levels

We used the full-length transcripts generated by the SMRT Iso-Seq analysis as reference sequences, and further combined with the short read dataset yielded from the Illumina sequencing platform to compare analysis by using Bowtie2 [26], then analyzed the expression level of UniGenes in the transcriptome of the sperm whale skin, which could be used for certain immune genes quantitative investigation. Briefly, the RESM software was used to calculate the expression levels of the UniGene expression values; all clean data generated by the Illumina sequencing platform were mapped back into the full-length transcripts as reference sequences to obtain the read count values of the isoforms in the skin transcriptome. To eliminate the influence caused by the difference in sequencing depth and transcript length, we converted all the read count values into FPKM values (the number of reads per kilobase length per million reads in the transcript) to calculate the expression of each isoform in the skin sample and then used the RPKM software for normalization [32].

To obtain a comprehensive functional annotation from the skin transcriptome of the sperm whale, we use Blast [33], Blast2GO [34], and InterProScan5 [35], after clustering and correcting to perform functional annotation with all transcriptome against seven different public protein and nucleotide databases, including the National Center for Biotechnology Information (NCBI) nonredundant protein sequence (Nr), NCBI nonredundant nucleotide sequence (NT), clusters of orthologous groups for complete eukaryotic genomes (KOG), Swiss-Prot, InterPro, Gene Ontology (GO), and Kyoto Encyclopedia of Genes and Genomes (KEGG) databases.

### 2.7. Sequence Analysis

TransDecoder [36] software was used to identify the candidate coding region in the transcripts. The longest open reading frames (ORFs) were extracted, and then the predicted coding sequences were verified by BLAST searches against the Swiss-Port database and by Hmmscan searches of the Pfam database to detect homologous protein sequences based on sequence similarity. The sperm whale NOD1 amino acid sequences were produced as a part of the PacBio full-length sequencing, which was uploaded into the NCBI database, the longest sequence named Isoform_6383 was used for analysis in the transcriptome (Accession ID: SRR13038369). We also retrieved other species’ NOD1 amino acid sequences from the NCBI database, whose accession IDs were provided as additional material (Table A1). The average length of those amino acid sequences was 957 aa. We used the MEGA X package to perform multiple sequence alignments on NOD1 of different species, then saved them in Fasta format, and used IQtree2 (IQ-TREE-2.0.6-windows) to construct the phylogenetic tree [37]. We chose the JTT + G4 model to construct the tree according to the Bayesian information criterion (BIC). Moreover, then the iTOL tool (https://itol.embl.de/(accessed on 4 February 2021)) was used to display, modify, and beautify the phylogenetic tree. Meanwhile, we also used the simple modular architecture research tool (SMART) to predict the conserved domains of NOD1 and RIP2 sequences based on their sequence homologies [38] and further confirmed them by BLAST conserved domain prediction.

## 3. Results

### 3.1. Full-Length Transcripts from the Skin of Sperm Whale

**Figure 2:**
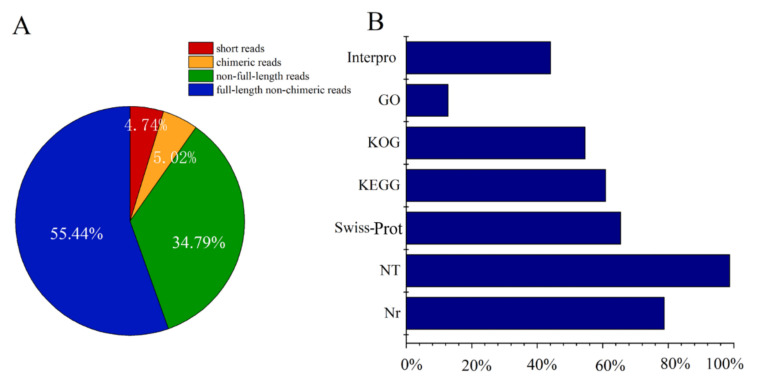
Skin sample PacBio sequencing results. (**A**) reads of insert (ROIs) classification: short reads (4.74%), chimeric reads (5.02%), non-full-length reads (34.795), and full-length non-chimeric reads (55.44%). (**B**) functional annotation of the full-length transcriptome in different databases: InterPro, Gene Ontology (GO), clusters of orthologous groups for complete eukaryotic genomes (KOG), Kyoto Encyclopedia of Genes and Genomes (KEGG), Swiss-Prot, NCBI nonredundant nucleotide sequence (NT), and nonredundant protein sequence (Nr).

To analyze the gene expression in the skin of the sperm whale, the extracted mRNA was processed to the full-length transcriptome using the PacBio Sequel platform. A total of 21.5 G subread bases was generated by two SMRT cells from the PacBio library. As a result, a total of 939,114,987 bp circular consensus sequences (CCS) (1,056,247 reads of insert) were obtained with a mean read length of 1776 bp and mean read quality of 0.93. All CCS reads were further classified into four categories, full-length non-chimeric (FLnc), chimeric, non-full-length (nFL), and short reads. In these categories, FLnc reads had the highest proportion, followed by nFL reads (Figure 2A). 187,767 nFL and 289,512 FLnc reads with a mean length of 1429 bp were simultaneously detected in tests containing 5′ adaptor sequences, 3′ adaptor sequences, and poly (A) tail signals. Only the FLnc and nFLreads were used in further analysis. The FLnc reads were clustered to form a consensus sequence using the ICE algorithm. For each cluster, if the FLnc and nFLreads coverage were sufficient, the Quiver program was run to refine the consensus. As a result, 129,174 high-quality consensus isoforms with accuracies > 99.91% were generated. The high-quality consensus in each library was merged by clustering and error correction, and the final full-length transcript was obtained by eliminating redundancy. A total of 96,350 full-length transcripts were generated with a mean length was 1705 bp, and the N50 length was 1996 bp (Table 1).

**Figure 3:**
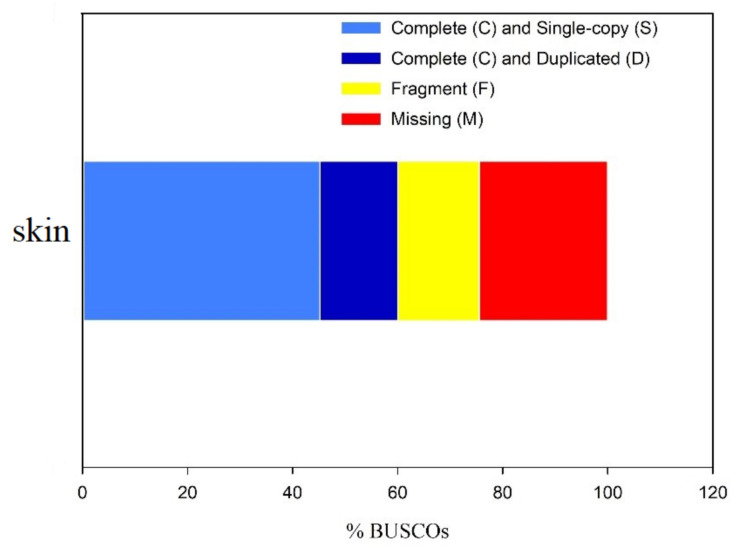
Benchmarking universal single-copy orthologs (BUSCO) assessment results of transcriptome generated by Illumine sequencing.

On the other hand, we also performed RNA-seq sequencing using RNA extracted from the skin tissue of the sperm whale, which generated 50.61 Mb raw reads. After filtering to remove low-quality data, 43.02 Mb clean reads were obtained. The clean reads were assembled and, after removing redundant reads, 35,982 UniGene with an N50 length of 2047 bp, GC content of 54.14%, and an average length of 1105 bp was obtained. The length of the specific distribution is shown in Table 2. The benchmarking universal single-copy orthologs (BUSCO) was used to evaluate the integrity of the transcriptome generated by Illumine sequencing [39]. The complete BUSCOs containing single-copy (S) and duplicated BUSCOs (D) accounted for 60.07% of all BUSCOs (Figure 3), and the fragment and missing occupied 15.51% and 24.42%, respectively. It suggested that the integrity of the transcriptome was reliable.

### 3.2. Annotations and Analysis of Full-Length Transcriptome

**Figure 4:**
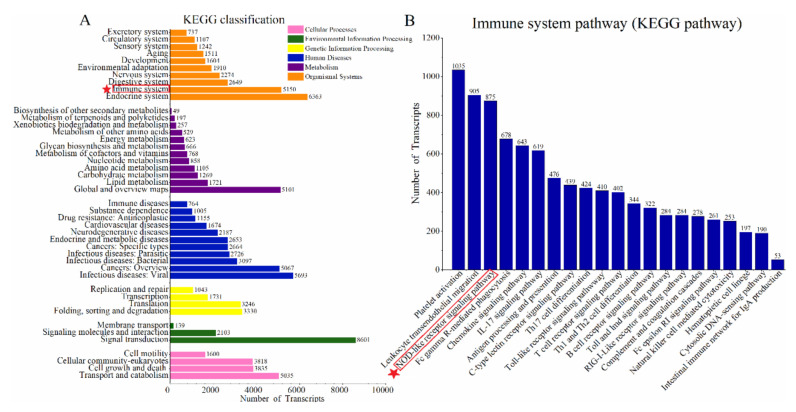
Analysis of full-length transcripts of the sperm whale. (**A**) Primary metabolic pathways annotated in the sperm whale full-length transcript in the KEGG database, and the immune system is framed by red star and rectangle; (**B**) the proportion of full-length transcripts annotated in the metabolic pathways of the immune system, and the nucleotide-binding oligomerization domain (NOD)-like receptor-signaling pathway on which we focused are marked by the red star and rectangle.

**Figure A1:**
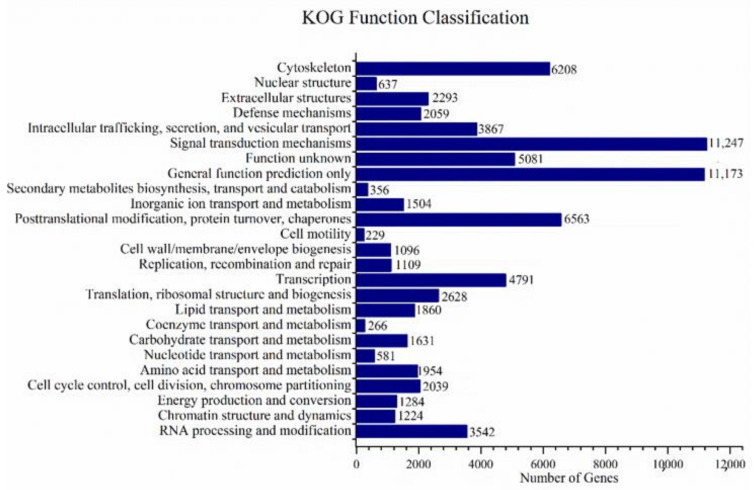
KOG annotation results of skin full-length transcriptome from a sperm whale.

Among the 96,350 full-length transcripts, 95,230 (98.84%) were annotated by homology searches against seven databases (Figure 2B). Among them, it was found that the annotation rates in Nr, NT, Swiss-Prot, KEGG, and KOG were relatively high, exceeding 50%, respectively. Through the functional annotation of genes and KEGG pathways analysis (Figure 4A), 58,661 transcripts were identified in the annotation pathways, and 342 different pathways were constructed, including chemokine-signaling pathway, cell pathway molecules, leukocyte transendothelial migration, and other pathways related to the metabolism of the immune system. The full-length transcripts were assigned to multiple KEGG pathways and KOG categories. The main representative pathways, such as focal adhesion, regulation of actin cytoskeleton, endocytosis, and phagosome, all belonging to the cellular processes, were classified into the level 1 process of KEGG categories. Moreover, those pathways were associated with molecular functions, including cell motility, cell communication, intracellular trafficking, secretion, and vesicular transport (Figure 4A and Figure A1, in Appendix A), suggesting that multiple cellular events were active in the skin of the sperm whale.

### 3.3. Enrichment of Immune-Related Pathways in the Skin of Sperm Whale

**Figure A2:**
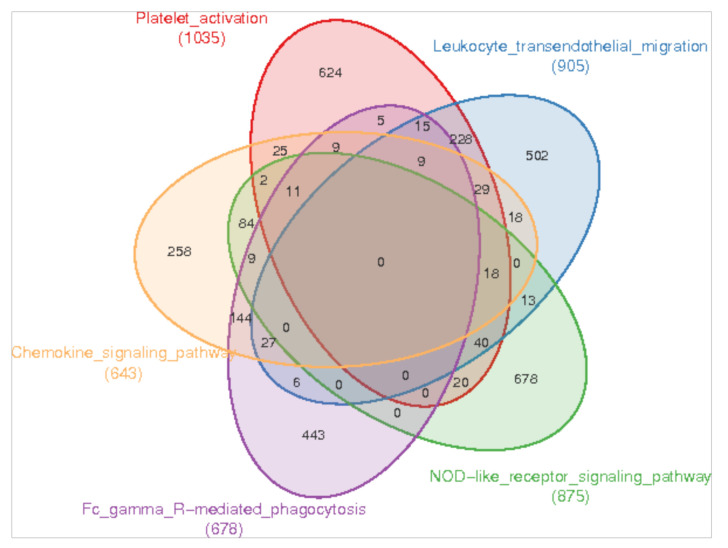
The number of overlapped transcripts among the top five immune pathways with the largest number of genes in KEGG annotation.

Because skin plays an important role in the regulation of the immune system, many transcripts of the sperm whale were annotated in immune-related pathways in the KEGG database, and then the immune-related pathways were analyzed., A total of 34,660 transcripts were annotated in KEGG pathways, and 20,476 transcripts overlapped in all KEGG pathways were annotated. Moreover, we also found that the top five immune pathways with the largest number of transcripts among the 21 immune pathways did not have overlapped transcripts (Figure A2, in Appendix A). A total of 5150 genes associated with 21 immune-related pathways were enriched (Figure 4A). Pathways associated with the higher number of genes were platelet activation (1035 genes) and leukocyte transendothelial migration (905 genes), followed by NOD-like receptor-signaling pathway (875 genes), Fc γ R-mediated phagocytosis (678 genes), chemokine-signaling pathway (643 genes), IL-17-signaling pathway (619 genes), antigen processing and presentation (476 genes), C-type lectin-receptor-signaling pathway (439 genes), Th17 cell differentiation (424 genes) and Toll-like receptor-signaling pathway (410 genes) and so on. These results roughly described the pathways related to immune function annotated in the sperm whale skin tissue.

### 3.4. NOD-Like Receptor Signaling Pathway

**Figure 5:**
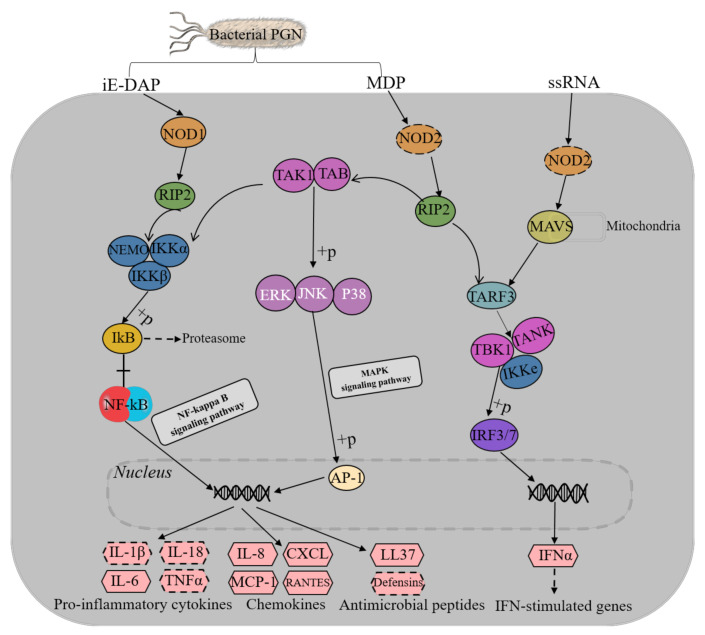
NOD-like receptor-signaling pathway constructed based on full-length sequencing and annotation results of the skin transcriptome of a sperm whale. Enzymes or proteins are frames in ellipses, immune-related products are framed in hexagon patterns, and the dotted hexagonal boxes represent substances that are not annotated in sperm whale skin transcriptome and metabolic pathways involved in the NOD-like receptor-signaling pathway are framed in rectangles. iE-DAP: γ-D-Glu-mDAP; MDP: muramyl dipeptide.

Based on the functional annotation and the metabolic pathway analysis of the skin transcriptome, 33 proteins coding genes related to the NOD-like receptor-signaling pathway in the skin of sperm whales were discovered, and the FPKM value relevant to each transcript was also listed (Table A2, in Appendix A). Combined with the analysis of KEGG comparison results, the high-quality transcript sequences involved in the NOD-like receptor-signaling pathway were blasted on NCBI, and it was found that these transcripts have a high degree of similarity with the corresponding protein-coding genes in the three toothed whale species (Table A2, in Appendix A). Meanwhile, based on the results of transcriptome annotation, we speculated that there was a NOD-like receptor-signaling pathway in the sperm whale (Figure 5). There were 33 protein-coding genes that were annotated in the NLR-signaling pathway in the sperm whale (Table A2, in Appendix A). After the NOD1 or NOD2 interacted with the bacterial PGN, RIP2 located downstream were further recruited and activated by interacting with the homotypic caspase recruitment domains (CARD) [40,41]. Subsequently, the IKK complex composed of Ikkβ, IKKα, and NEMO was indirectly activated, leading to degradation of the NF-κB inhibitor IkBα, translocation of NF-κB into the nucleus, and inducing the transcription of chemokines and other substances. In addition to activating the NF-κB pathway, NOD2 stimulation can also lead to the activation of MAPKs (ERK, JNK, P38). Both NF-κB and MAPK pathways can induce the secretion of proinflammatory cytokines and chemokines and the production of antimicrobial peptides. Furthermore, the NOD2 receptor can recognize single-strand RNA (ssRNA), activate IRF3 indirectly through the mitochondrial antiviral-signaling protein (MAVS) signal, leading to the generation of IFNα, an of type I IFNs.

### 3.5. Structural and Evolutional Analysis of NOD1 Gene in Sperm Whale

**Figure 6:**
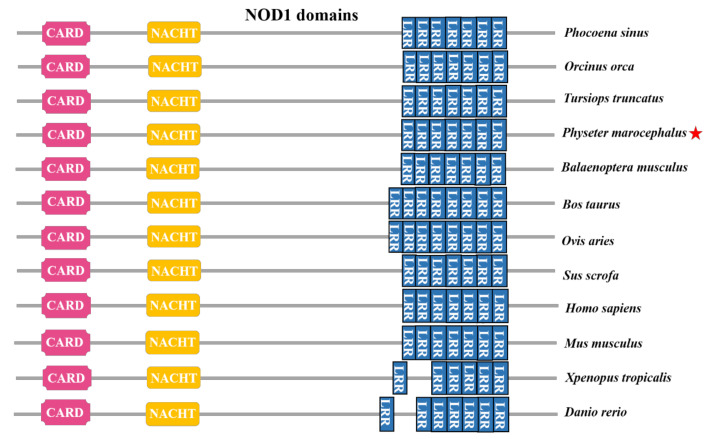
Schematic representation of the conservative domains of the NOD1 sequences in some different typical vertebrate species. The sperm whale is highlighted by a red star. The domains were identified by using simple modular architecture research tool (SMART). CARD: caspase recruitment domain; NACHT: nucleotide-binding/oligomerization domain; LRR: leucine-rich repeats.

As for the sperm whale, in terms of the encoded structural-functional domains of the *NOD1* gene (Figure 6), the *NOD1* genes from different species share many common structural-features, all possessing C-terminal leucine-rich repeat (LRR) domains and a central nucleotide-binding-oligomerization domain (NBD domain), which is NACHT domain, and N-terminal CARD domains.

**Figure 7:**
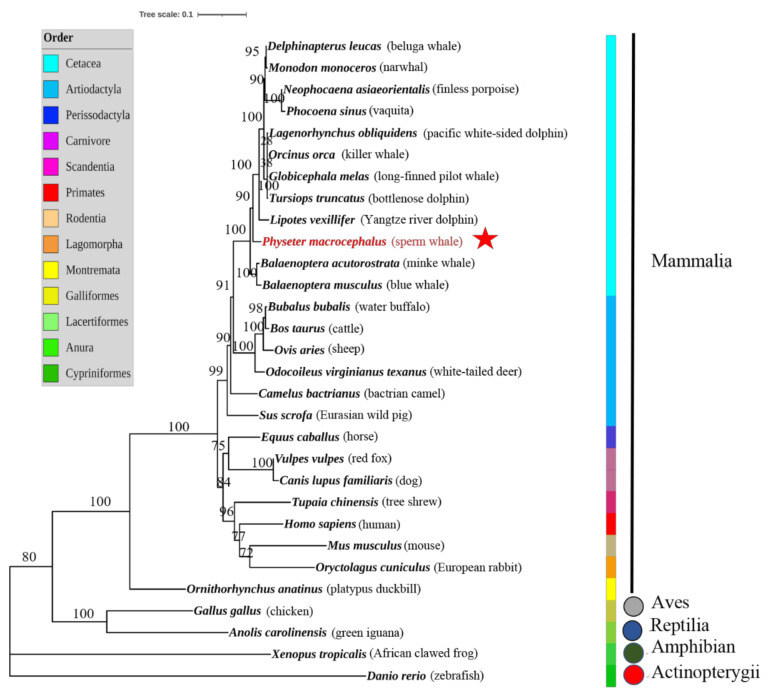
The phylogenetic tree of the NOD1 protein sequences was constructed using JTT + G4 model based on the Bayesian information criterion in IQ-TREE. The node number represented the bootstrap confidence level of the Bayesian analysis. The five classes were Mammalia, Aves, Reptilia, Amphibia and Actinopterygii, respectively. The sperm whale (*P. marocephalus*) is highlighted in red and marked with a red star on the node. Meanwhile, *Danio rerio* was chosen as the out-group.

The NOD1 amino acid sequences were aligned with 30 different species from five classes, including Mammalia, Aves, Reptilia, Amphibia, and Actinopterygii, to construct the phylogenetic tree. The NOD1 sequence of the sperm whale clustered with orthologous sequences of other cetacean species to form a group in which the sperm whale sequence’s NOD1 was closest to that of the Yangtze river dolphin (*Lipotes vexillifer)* (Figure 7). As we expected, compared with the other species among all the five classes, the cetacean NOD1 protein orthologous formed a cluster and was closely related to its terrestrial relative water buffalo (*Bubalus bubalis*) in order Artiodactyla.

## 4. Discussion

In the process of cetaceans returning from land to the sea, the change of pathogenic microorganisms poses a severe challenge to their survival and may drive the evolution and adaptation of immune genes [42]. Skin is not only one of the gateways between cetacean and the external water environment but also the first barrier for defense and plays an important role in defending against pathogens [43]. The dominant cell type in the cetacean epidermis is lipokeratinocyte, which helps the mechanical strength, buoyancy, and insulation of cetacean skin [44]. In addition to as the physical barrier, cetacean skin can also detoxify chemicals that pass through the stratum corneum by xenobiotic pathways [45]. Moreover, the skin is also an immune organ. It has shown that proinflammatory cytokines can induce the production of β-defensins, which may serve as a nonspecific defense against bacteria, fungi, and algae [46]. By analyzing the selective pressure of immune-related genes involved in the TLR-signaling pathway in cetacean’s innate immune systems, it had found that those genes in the TLR-signaling pathway were under selective pressure, suggesting the cetacean’s immune system has adapted to the pathogenic microorganisms during their transition from the terrestrial to the marine ecosystem [20]. However, the molecular pathway of the innate immune remains unknown in sperm whales. In this study, the short reads sequenced by RNA-Seq and the full-length transcripts generated by PacBio Iso-Seq of the sperm whale’s skin were obtained and used to investigate immune-related pathways in sperm whale skin tissue. The proportion of the complete BUSCOs containing a single-copy (S) and duplicated BUSCO (D) was more than 60% of the total BUSCO, which was similar to other studies [47,48,49]. Moreover, many genes generated by full-length transcriptome were annotated with protein-related pathways, such as transcription, amino acid transport, and metabolism (Figure A1; in Appendix A), indicating the participation of these biological processes could be the basis of the protein biosynthesis and secretion of the sperm whale skin. Those data would provide a certain data basis for the future research of sperm whale immune research and metabolic pathways.

Because there are more pathogenic organisms such as bacteria, fungi, and parasites in aquatic environments than on land [9,10], the immune mechanisms or immune substances of aquatic organisms have recently attracted many interests [50,51,52]. As the first line of defense, the innate immune system can detect and remove harmful microbes. Although aquatic organisms are more exposed to microorganisms than land organisms, they may develop a series of strategies to deal with huge environmental pressure. For example, fish majorly relied on innate immunity during their developmental stages [53].

As a group of aquatic mammals, cetaceans may have also evolved a better innate immune system. Innate immunity involves a family of proteins recognizing the microbial called pattern recognition receptors (PRRs) [54]. As the first class of cellular PRRs to be identified, TLRs were a class of extracellular transmembrane PRRs [55]. They have been studied widely in many animals, including fish, chicken, and humans [53,56], even in cetaceans [57]. The NLRs (NOD-like proteins) are important intracellular cytoplasmic PRRs that have been investigated in immunity, inflammation, and disease. In previous studies, the roles of the NLRs family in immune mechanisms had been well studied in humans, mice, and fish [52,58,59]; however, the NLR pathway has not been reported in any cetaceans until now.

In this study, based on the gene annotation and the metabolic pathway analysis of the skin transcriptome, the coding genes annotated the NLR-signaling pathway were blasted on NCBI, the transcripts were found to have very high consistency with sequence from the sperm whale RefSeq genome. Meanwhile, the NLR-signaling pathway was studied in the other species (human, mice, and fish) [53,60,61], it was speculated that the NLR-signaling pathway was extremely likely to be annotated in the sperm whale. The NOD1/NOD2 recognized the bacterial PGN and then transmitted and stimulated immune signals, which generated several central immune factors, immune substances, and antimicrobial peptides that played significant roles in the body’s immune responses (Figure 5). Unlike in the other mammals studied, in the sperm whale skin, *NOD2* was not annotated with the NOD-like receptor-signaling pathway in our sample, maybe because of the low expression. Otherwise, the *NOD2* expression was commonly found in the intestine, lung, and oral cavity [22].

In the NOD-like receptor-signaling pathway, NOD1 and NOD2 were both the important proteins that recognized PGNs through their LRR domains [62]. Bacterial PGN was associated with NOD1 and NOD2, then would recruit and interact with RIP2, leading to downstream-signaling events that eventually induced the NF-κB and MAP kinase activation (Figure 6). NOD2 cannot only recognize bacterial PGN (MDP) but also combine with single-stranded RNA (ssRNA) to trigger a series of immune-signaling pathways and produce some immune factors to resist the invasion of pathogens [63,64] (Figure 5). NOD1 and NOD2 can also affect the abundance levels of immune substances such as chemokines and cytokines, which are important mediators in the immune response, and their abundance levels directly affected the occurrence and regulation of immune effects [65].

**Figure A3:**
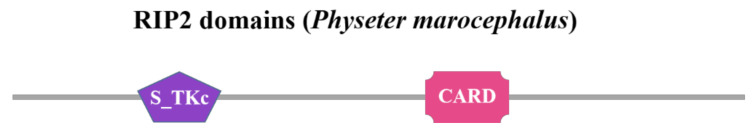
Schematic diagram of RIP2 domains in the sperm whale. S_TKC: serine/threonine protein kinase, catalytic domain; CARD: caspase recruitment domain.

RIP2 is a specific serine/threonine kinase activity that is a member of the RIP family [66,67]. From our annotation, RIP2 (Isoform_25948, Accession ID: SRR13038369) in the sperm whale contained an N-terminal kinase domain and a CARD domain that could interact with other proteins containing CARD domain (Figure A3, in Appendix A), such as NOD1 or NOD2 [40,41]. As shown in Table A2 (in Appendix A), the FPKM expression of RIP2 was about 0.71–1.52. RIP2 has been shown to play a vital role in NOD1- and NOD2-mediated innate immune responses. Moreover, RIP2 deficiency was closely related to cellular-signaling and cytokine responses in the NLR-signaling pathway [68,69,70]. The responses of RIP2-deficient and kinase-dead mice to stimulation with the NOD1 and NOD2 ligands in vitro, which suggested that neither the NLR-mediated inflammatory chemokines nor cytokines could be yield normally in the absence of RIP2 or loss of RIP2 kinase activity, further demonstrating the importance of RIP2 kinase in maintaining the inflammatory immune response [71]. Moreover, RIP2 overexpression was shown to mediate the phosphorylation and activation of TAK1, which is involved in the NLR-mediated-signaling pathway [72].

NF-κB is a highly conserved transcription factor in evolution, which is widely present in various tissues. Many pathogens and viruses can activate NF-κB, indicating that NF-κB is an evolutionarily conserved mediator of immune and inflammatory [73,74]. The NF-κB family is mainly composed of five family members: c-REL, RELB, NF-κB1 (p105/p50), NF-κB2 (p100/p52) and RELA (p65) [75]. NF-κB proteins are a type of homologous/heterologous nuclear transcription factor, which is formed by members of the NF-κB family and plays an important role in cells. It has shown that p50 and p65 can form heterodimer NF-κB existing in the human placenta [76]. In our transcriptome, the genes encoding RELA and NFKB1 proteins were annotated, their FPKM expressions ranges are about 0.08–10.86, 1.34–3.73, respectively (Table A2, in Appendix A), and by blasting, the RELA is the p65 subunit of NF-κB, and the NFKB1 is p105, which plays the role as a precursor for p50 and can be translated into p50 [77]. We speculated that NF-κB might also exist in the skin of the sperm whale as a heterodimer composed of p65-p50, which was worthy of further verification in the future. NF-κB played an essential role in the immune response. Most of the NF-κB dimers can bind to the IKB inhibitor that remains in the cytoplasm before the IKK complex activated IKB phosphorylation [78]. Intestinal invading bacteria can activate NF-κB in human intestinal epithelial cells, leading to the production of inflammatory factors, such as IL-8, MCP-1, and TNF-α that are vital components of the inflammatory, immune, and stress response [79]. In addition to the NLR-signaling pathway, NF-κB also plays a similar key role in TNF-signaling pathways by producing important-signaling molecules to mediate cell proliferation and death [80].

Most NOD-family members have a tripartite domain architecture comprising an N-terminal effector-binding domain (EBD), a centrally located NOD domain, and a C-terminal ligand-recognition domain (LRD). The EBD interacts with downstream effector molecules following activation of the-signaling cascade. In the NOD proteins, the EBD of NOD proteins is structurally variable and divided mainly into the caspase recruitment (CARD) domain and the pyrin (PYD) domain [81]. The EBD in the NOD1 proteins is a CARD domain. The centrally located NOD domain is responsible for ATPase activity and induces self-oligomerization, and which is classified as a NACHT domain in NOD1. The LRR domain recognized exogenous and endogenous ligands. When the small peptides derived from PGNs were released into the cytoplasm, they would be recognized and bound by the LRR domain of NOD1 [69], then NOD1 initiated RIP2 through CARD–CARD domain interactions [40] and subsequently mediated the activation of NF-κB (Figure 5).

In this study, the NOD1 structure of the sperm whale and other different species were compared. The amino acid sequence of NOD1 from the sperm whale was highly similar to the other orthologous sequences of other vertebrates, especially the highly conserved CARD, NATCH, and LRR domains (Figure 6). The structure of the NOD1 domains was very similar in the cetacean species and also similar to the NOD1 domains in *Bos taurus* in order Artiodactyla. In particular, there were only a few differences that the number of leucine-rich repeats in the LRR domains of the sperm whale NOD1 was one less than the number in *B. taurus* and *Ovis aries* (Figure 6). We also used the NOD1 full-length sequence of the sperm whale to construct a phylogenetic tree (Figure 7). The sperm whale NOD1 protein formed a cluster with the orthologous proteins of other cetaceans and was closely related to its terrestrial relative *B. bubalis* in order Artiodactyla. From the structure and phylogenetic analysis, we presume the function of NOD1 is conserved in the sperm whale and even in other cetaceans, implying it may play a vital role in the immune process of the sperm whale.

## 5. Conclusions

A total of 96,350 full-length transcripts with an average length of 1705 bp were generated, and 5150 genes were detected to associate with 21 immune-related pathways by gene annotation enrichment analysis. Moreover, 89 encoding genes corresponding to 33 proteins related to the NOD-like receptor-signaling pathway of innate immunity were discovered in the skin of the sperm whale. By sequence comparison analysis, it revealed that these proteins coding genes have a high consistency with the sequences that come from a RefSeq genome of the sperm whale, which was a deduced transcriptome in NCBI, and those were also highly consistent with the sequences of *L.vexillifer* and *T. truncatus.* We also found that *NOD1*, *RIP2,* and *NF-κB* existed in the NOD-like receptor-signaling pathway, which was proved to play important roles in resisting the invasion of pathogens in many other species. Furthermore, we speculated that the function and domains of NOD1 of the sperm whale were highly conserved by structure and phylogenetic analysis with orthologs in other vertebrates. Our results provide information about the NLR-signaling pathway in the skin of the sperm whale and deepen our understanding of the innate immune process of the sperm whale, a secondarily adapted marine mammal.

## Figures and Tables

**Table 1:** The final transcripts obtained by PacBio sequencing.

Sample	Read of Insert	Total Transcripts	Mean Length (bp)	N50 (bp)
Total	1,056,247	96,350	1705	1996

**Table 2:** Length distribution of gene sequences in the spliced transcripts.

Sequence Size (bp)	UniGene Number
200–500	16,252
500–1000	7251
1000–2000	6444
2000–3000	3192
≥3000	2843
Total	35,982

## Data Availability

Transcriptome sequences were deposited to the NCBI database, the BioProject ID containing the SRA data were PRJNA67782 and PRJNA676103.

## Appendix A

**Table A1:** A list of NOD1 gene information of 28 different species used in the phylogenetic tree.

Species Name	Protein ID	Length (Amino Acids)
*Tursiops truncatus*	XP_019781619.1	953
*Lagnorhynchus obliquidens*	XP_026958076.1	953
*Globicephla melas*	XP_030713315.1	953
*Orcinus orca*	XP_033284214.1	953
*Phocoena sinus*	XP_032499084.1	972
*Neophocaena asiaeorientalis*	XP_024610766.1	963
*Monodon monoceros*	XP_029089062.1	953
*Delphinapterus leucas*	XP_022439858.1	953
*Lipotes vexillifer*	XP_007466161.1	953
*Physeter macrocephalus*	Isoform_6383	958
*Odocoileus virginianus texanus*	XP_020748129.1	954
*Balaenoptera acutorostrata*	XP_028020573.1	818
*Balaenoptera musculus*	XP_036718877.1	953
*Ovis aries*	XP_004007979.2	954
*Bubalus bubalis*	XP_006055719.1	954
*Bos taurus*	XP_005205565.2	954
*Camelus bactrianus*	XP_010969658.1	953
*Sus scrofa*	NP_001107749.1	953
*Anolis carolinensis*	XP_003222248.2	950
*Equus caballus*	XP_023495227.1	897
*Tupaia chinensis*	ELW67927.1	1109
*Homo sapiens*	AAD28350.1	953
*Oryctolagus cuniculus*	XP_002713781.1	953
*Mus musculus*	AAN52479.1	953
*Ornithorhynchus anatinus*	XP_001512159.2	950
*Gallus gallus*	NP_001305367.1	951
*Danio rerio*	XP_002665106.3	940
*Xenopus tropicalis*	XP_031759856.1	945
*Canis lupus familiaris*	XP_013974568.1	964
*Vulpes vulpes*	XP_025874889.1	953

**Table A2:** Proteins transcripts that were associated with NOD-like receptor-signaling pathway annotated in the skin transcriptome of the sperm whale.

Proteins/Enzymes Name	Genes ID in This Transcriptome	*** FPKM (Min–Max)	Sequence Alignment Identity Percentage (%)		
			*Physeter macrocephalus*	*Lipotes vexillifer*	*Tursiops truncatus*
Nucleotide-binding oligomerization domain-containing protein 1 (NOD1)	Isoform_6383 Isoform_44218 Isoform_24661 Isoform_40056	0.41–7.56	100.00 (XP_023984584.1) 100.00 (XP_023984584.1) 100.00 (XP_023984584.1) 92.05 (XP_023984584.1)	96.41 (XP_007466161.1) 98.94 (XP_007466161.1) 98.52 (XP_007466161.1) 90.73 (XP_007466161.1)	96.73 (XP_019781619.1) 97.88 (XP_033719017.1) 97.78 (XP_019781638.1) 90.07 (XP_033719017.1)
Interleukin 6(IL-6)	Isoform_60901 Isoform_23835	1.32–1.59	100.00 (XP_007105488.3) 100.00 (XP_007105488.3)	NM NM	100.00 (XP_019778462.1) 100.00 (XP_019778462.1)
Interleukin 8 (IL-8)	Isoform_34284	2.71	100.00 (XP_007126317.1)	98.02 (XP_007467625.1)	96.04 (NP_001267571.1)
C–X–C motif chemokine 2 (CXCL2) C–X–C motif chemokine 3 (CXCL3)	Isoform_67128 Isoform_70740 Isoform_34284 Isoform_69403 Isoform_45444	0.79–7.18 2.29-3.46 2.29-3.46	98.53 (XP_023971193.1) 98.53 (XP_023971193.1) 98.44 (XP_023971204.1) 98.41 (XP_007126322.2) 98.44 (XP_007126322.2)	96.88 (NP_007463281.1) 96.88 (NP_007463281.1) 96.88 (NP_007463281.1) NM NM	94.12 (NP_004331677.2) 94.12 (NP_004331677.2) 93.75 (NP_004331677.2) NM NM
C–C motif chemokine 2 (MCP-1)	Isoform_85283 Isoform_14905 Isoform_68475	0.08–3.03	100.00 (XP_007467625.1) 91.11 (XP_023983766.1) 98.44 (XP_023983766.1)	90.36 (XP_007458436.1) 82.22 (XP_007458436.1) 89.80 (XP_007458436.1)	NM NM NM
Cathelicidin antimicrobial peptide (LL37)	Isoform_27979 Isoform_90183 Isoform_92246	0.11–15.39	100.00 (XP_007101317.2) 100.00 (XP_007101317.2) 100.00 (XP_007101317.2)	90.91 (XP_007446211.1) 89.94 (XP_007446211.1) 77.99 (XP_007446211.1)	93.94 (XP_033720747.1) 88.89 (XP_033720747.1) 90.35 (XP_033720747.1)
transcription factor AP-1 (AP-1) Interferon α (IFNα)	Isoform_38241 Isoform_63047 Isoform_56853 Isoform_75661	0.25–264.37 1.04	100.00 (XP_023975690.1) 100.00 (XP_023975690.1) 80.97 (XP_023975690.1) 100.00 (XP_023982688.1)	100.00 (XP_007462636.1) 100.00 (XP_007462636.1) NM 58.90 (XP_007455007.1)	100.00 (XP_019797942.2) 100.00 (XP_019797942.2) NM 97.22 (AFH89765.1)
Receptor-interacting serine/threonine–protein kinase 2 (RIP2)	Isoform_20477 Isoform_25948 Isoform_60840 Isoform_78887	0.71–1.52	100.00 (XP_023982836.1) 100.00 (XP_023982836.1) 100.00 (XP_023982837.1) 100.00 (XP_023982837.1)	98.33 (XP_007446375.1) 98.33 (XP_007446375.1) 97.07 (XP_007446375.1) 98.39 (XP_007446375.1)	97.95 (XP_033699058.1) 97.95 (XP_033699058.1) 96.31 (XP_033699060.1) 98.39 (XP_033699059.1)
transcription factor p65(RELA)	Isoform_11257 Isoform_15246 Isoform_14067 Isoform_35442	0.08–10.86	100.00 (XP_023989099.1) 87.06 (XP_023989099.1) 99.64 (XP_007128247.1) 100.00 (XP_007128247.1)	96.01 (XP_007462051.1) 87.06 (XP_007462053.1) 96.01 (XP_007462052.1) 94.46 (XP_007462051.1)	NM NM NM NM
Nuclear factor NF-kappa-B p105 subunit (NFKB1)	Isoform_2285 Isoform_78940	1.34–3.73	100.00 (XP_023982362.1) 100.00 (XP_023982361.1)	95.21 (XP_007463876.1) 97.52 (XP_007463874.1)	91.78 (XP_019782050.1) 96.88 (XP_019782049.1)
Inhibitor of nuclear factor kappa-B kinase subunit β (Ikkβ)	Isoform_16029 Isoform_3742	0.64–2.16	99.44 (XP_028341955.1) 99.73 (XP_028341955.1)	97.32 (XP_007455533.1) 98.02 (XP_007455533.1)	96.78 (XP_033704813.1) 98.02 (XP_033704810.1)
Inhibitor of nuclear factor kappa-B kinase subunit α (Ikkα)	Isoform_69638 Isoform_28559	0.25–3.37	100.00 (XP_028337608.1) 100.00 (XP_028337608.1)	100.00 (XP_007117682.1) 97.84 (XP_007470397.1)	100.00 (XP_033697982.1) 99.27 (XP_033697983.1)
NF-kappa-B inhibitor α (NFKBIA)	Isoform_44406 Isoform_62842 Isoform_57121 Isoform_5319	0.03–66.12	100.00 (XP_007119015.1) 100.00 (XP_007119015.1) 100.00 (XP_007119015.1) 89.70 (XP_007119015.1)	95.54 (XP_007464888.1) 93.87 (XP_007464888.1) 95.37 (XP_007464888.1) NM	NM NM NM NM
NF-kappa-B inhibitor β(NFKBIB)	Isoform_64970 Isoform_49218 Isoform_61453 Isoform_30066	0.36–16.21	100.00 (XP_007114641.1) 99.46 (XP_007114641.2) 100.00 (XP_007114641.2) 100.00 (XP_007114641.2)	98.58 (XP_007464742.1) 97.27 (XP_007464742.1) 99.08 (XP_007464742.1) 98.71 (XP_007464742.1)	98.58 (XP_019796701.1) 97.28 (XP_019796701.1) 98.18 (XP_019796701.1) 98.39 (XP_019796701.1)
Mitogen-activated protein kinase kinase kinase 7 (TAK1)	Isoform_11038 Isoform_3956 Isoform_6441	0.39–2.85	100.00 (XP_007126290.1) 100.00 (XP_007126290.1) 100.00 (XP_007126290.1)	NM NM NM	NM NM NM
TAK1-binding protein 3(TAB3)	Isoform_24688	1.38	99.81 (XP_023972426.1)	98.85 (XP_007456911.1)	NM
Mitogen-activated protein kinase 3(ERK)	Isoform_43270 Isoform_52694 Isoform_78038	2.06–23.34	NM NM NM	98.87 (XP_007452842.1) 100.00 (XP_007452842.1) 100.00 (XP_007452842.1)	NM NM NM
Mitogen-activated protein kinase 9 (MAPK9) (JNK)	Isoform_51119 Isoform_55328	0.43–1.36	100.00 (XP_023972057.1) 99.57 (XP_023972058.1)	99.59 (XP_007465571.1) 99.13 (XP_007465571.1)	NM NM
Mitogen-activated protein kinase 8 (MAPK8) (JNK)	Isoform_76485	0.05	NM	98.82 (XP_007446292.1)	NM
P38 MAP kinase 11 (P38 β) P38 MAP kinase 12 (P38 γ) P38 MAP kinase 13 (P38 δ)	Isoform_54378 Isoform_28186 Isoform_36890 Isoform_46157 Isoform_9786 Isoform_5589 Isoform_11611 Isoform_12832 Isoform_13409	1.77 0.36–3.83 0.06–20.27	75.34 (XP_023983249.1) 100.00 (XP_028346617.1) 59.13 (XP_028346617.1) 96.18 (XP_028346617.1) 100.00 (XP_023977640.1) 100.00 (XP_023977640.1) 95.60 (XP_023977640.1) 100.00 (XP_023977640.1) 100.00 (XP_023977640.1)	73.97 (XP_007454143.1) 97.90 (XP_007453950.1) 56.15 (XP_007467328.1) 94.24 (XP_007453950.1) 96.06 (XP_007467328.1) 96.17 (XP_007467328.1) 93.41 (XP_007467328.1) 96.06 (XP_007467328.1) 94.17 (XP_007467328.1)	NM 98.51 (XP_033721676.1) 56.92 (XP_019805222.1) 94.96 (XP_033721674.1) 95.07 (XP_004313866.1) 95.08 (XP_004313866.1) 92.31 (XP_004313866.1) 95.07 (XP_004313866.1) 95.15 (XP_019805222.1)
Mitochondrial antiviral-signaling protein (MAVS)	Isoform_11457 Isoform_8429	0.19–1.83	NM NM	94.08 (XP_007454896.1) 94.08 (XP_007454896.1)	92.91 (XP_033695234.1) 92.01 (XP_033695233.1)
TAK1-binding protein 3 (TAB3)	Isoform_24688	0.99	99.81 (XP_023972426.1)	98.85 (XP_007456911.1)	NM
Inhibitor of nuclear factor kappa-B kinase subunit epsilon (IKKe)	Isoform_49506 Isoform_42897 Isoform_44117 Isoform_39392 Isoform_73428	0.51–1.29	NM NM NM NM 100.00 (XP_028342232.1)	78.84 (XP_007470765.1) 95.02 (XP_007470765.1) 93.63 (XP_007470765.1) 94.96 (XP_007470765.1) 94.32 (XP_007470765.1)	NM NM NM NM NM
TRAF family member-associated NF-kappa-B activator (TANK)	Isoform_44877 Isoform_30925 Isoform_67916 Isoform_35515	0.03–1.95	NM 98.86 (XP_007107800.2) 100.00 (XP_028334198.1) 100.00 (XP_028334197.1)	96.77 (XP_019778469.1) 96.80 (XP_019778470.1) 96.77 (XP_007457066.1) 95.12 (XP_007457065.1)	NM 94.65 (XP_019778476.1) 97.42 (XP_019778476.1) 95.89 (XP_019778469.1)
Interferon regulatory factor 3 (IRF3)	Isoform_25349 Isoform_45963 Isoform_65550 Isoform_73641 Isoform_51357	0.6–3.42	93.54 (XP_007112499.1) 100.00 (XP_007112499.1) 100.00 (XP_007112499.1) 95.52 (XP_007112501.1) 100.00 (XP_007112498.1)	87.76 (XP_007463434.1) 94.78 (XP_007463434.1) 95.08 (XP_007463433.1) 96.22 (XP_007463435.1) 95.93 (XP_007463434.1)	87.41 (XP_033701337.1) NM95.04 (XP_033701337.1) 84.82 (XP_033701337.1) 95.69 (XP_033701337.1)
Interferon regulatory factor 7 (IRF7) Inhibitor of nuclear factor kappa-B kinase subunit γ (NEMO) C–C motif chemokine 5 (CCL5/RANTES) TNF receptor-associated factor 3 (TRAF3)	Isoform_16065 Isoform_57550 Isoform_32564 Isoform_44904 Isoform_30772 Isoform_32236 Isoform_33819 Isoform_86381 Isoform_14574	0.4–1.62 0.13–2.14 0.85 0.1	100.00 (XP_028341713.1) 87.47 (XP_028341711.1) 87.47 (XP_028341711.1) 100.00 (XP_028341713.1) NM NM NM 100.00 (XP_028355421.1) NM	95.04 (XP_007465980.1) 84.88 (XP_007465980.1) 93.44 (XP_007465980.1) 86.14 (XP_007465980.1) 92.79 (XP_007457510.1) 93.45 (XP_007457510.1) 93.45 (XP_007457510.1) 96.83 (XP_007447107.1) 93.04 (XP_007455372.1)	96.17 (XP_033717457.1) NM95.29 (XP_033717454.1) 95.32 (XP_033717457.1) 92.48 (XP_033705598.1) 93.45 (XP_033705598.1) 93.45 (XP_033705598.1) 95.24 (XP_004322904.1) 94.39 (XP_019776547.2)

*** FPKM: the FPKM value of the transcripts annotated in NOD-like receptor signaling pathway of the sperm whale.
